# 分散固相萃取-高效液相色谱-串联质谱法测定消毒产品中42种抗微生物药物

**DOI:** 10.3724/SP.J.1123.2024.02018

**Published:** 2024-11-08

**Authors:** Jiaqi TONG, Guhuan ZHU, Hangzhen LAN, Honglan SHI, Xiaoyan ZHU, Yao SUN

**Affiliations:** 1.宁波海关技术中心, 浙江 宁波 315000; 1. Ningbo Customs Technology Center, Ningbo 315000, China; 2.宁波中盛产品检测有限公司, 浙江 宁波 315000; 2. Ningbo Joysun Product Testing Service Company, Ningbo 315000, China; 3.宁波大学食品科学与工程学院, 浙江 宁波 315000; 3. College of Food Science and Engineering, Ningbo University, Ningbo 315000, China; 4.大连海关技术中心, 辽宁 大连 116000; 4. Technological Center of Dalian Customs, Dalian 116000, China

**Keywords:** 消毒产品, 抗微生物药物, 高效液相色谱-串联质谱, 分散固相萃取, disinfection products, antimicrobials, high performance liquid chromatography-tandem mass spectrometry (HPLC-MS/MS), dispersive solid phase extraction

## Abstract

本研究采用分散固相萃取-高效液相色谱-串联质谱(HPLC-MS/MS)测定膏霜、凝胶和水剂等基质类型的消毒产品中42种抗微生物药物(7种磺胺类、10种喹诺酮类、3种林可酰胺类、5种四环素类、3种大环内酯类、8种唑类、3种嘌呤核苷类、1种呋喃类、1种非多烯类及1种类固醇类药物)含量的测定方法。消毒产品样品用水辅助分散,用0.5%甲酸乙腈溶液提取,用EMR-Lipid吸附剂除去提取液中长链烃类化合物等样品基质成分。选用Poroshell 120 EC-C18色谱柱(150 mm×3.0 mm, 2.7 μm)分离,以0.1%甲酸乙腈溶液和0.1%甲酸水溶液为流动相,梯度洗脱。选用电喷雾离子源,正离子(ESI^+^)扫描模式和多反应监测(MRM)模式检测,保留时间和选择离子定性,外标法定量。在优化的条件下,42种抗微生物药物可以有效分离,基质效应对目标化合物的影响较弱。在0.25~5.0 mg/kg范围内,42种目标化合物线性良好,相关系数(*r*)>0.99。以色谱峰信噪比(*S/N*)≥3评估检出限(LOD),在取样量为0.2 g、定容体积为10 mL时,3种基质中42种抗微生物药物的检出限为0.03~0.10 mg/kg。在0.25、1.0和5.0 mg/kg 3个添加水平下,膏霜、凝胶和水剂3种基质中42种抗微生物药物的平均回收率为80.3%~109.8%,相对标准偏差(RSD)≤9.8%。采用本方法检测市售消毒产品中抗微生物药物,共检出2批阳性样品。所建立的方法操作简便,准确性好,精密度高,可适用于膏霜、凝胶和水剂等类型消毒产品中42种抗微生物药物的快速测定。

## Abstract (en)

Antimicrobials inhibit the growth and reproduction of microorganisms thereby alleviating skin and other problems caused by microorganisms. Antimicrobials are classified into different categories, including antibacterials, antifungals, and antivirals, among others, and include azoles, sulfonamides, tetracyclines, quinolones, and many other classes of synthetic and natural compound. The inappropriate or excessive use of antimicrobials can damage skin and other human organs and increase antimicrobial resistance. Relevant regulations and standards clearly state that antimicrobials are prohibited for use as ingredients in disinfection products. However, since antimicrobials enhance the disinfection or antibacterial effect of a product, with a significant short-term effect, antimicrobials are occasionally illegally added to disinfectant products, including those intended for human use. Therefore, establishing testing methods that provide technical support for enforcing regulations is an urgent objective.

Herein, a method was established for the analysis of 42 antimicrobials in disinfection products, that is applicable to common types of disinfection-product matrix, including creams, gels, and aqueous solutions, using high performance liquid chromatography-tandem mass spectrometry (HPLC-MS/MS) combined with dispersive solid phase extraction. The 42 antimicrobials comprise antibacterials, antifungals, and antivirals, and include seven sulfonamides, ten quinolones, three lincosamides, five tetracyclines, three macrolides, eight azoles, three purine nucleoside analogs, one furan, one nonpolyene antifungal, and one steroid. Briefly, 0.2 g of a sample was first dispersed in 2 mL of water and then extracted with 10 mL of 0.5% formic acid in acetonitrile, with 3 g of anhydrous Na_2_SO_4_ added to remove water. After centrifugation, 5 mL of the supernatant was cleaned using dispersive solid phase extraction with EMR-Lipid as the adsorbent. Lipids, waxes, surfactants, and moisturizing lubricants are commonly used as cream and gel matrices. Matrix substances containing long carbon chains dissolved in acetonitrile were removed using the EMR-Lipid adsorbent. Target analytes were separated on a Poroshell 120 EC-C18 analytical chromatography column (150 mm×3.0 mm, 2.7 μm), with 0.1% formic acid in acetonitrile and 0.1% formic acid aqueous solution used as mobile phases under gradient-elution conditions. The target analytes in the test solution were detected in positive ionization (ESI^+^) and multi-reaction-monitoring (MRM) modes. Analytes were characterized in terms of their retention times and selected ions, and quantified using the external-standard method. The main factors affecting method response, recovery, and sensitivity, such as the extraction method and solvent, purification method and adsorbent, mobile phase, and MS conditions, were examined during sample pretreatment and instrumental analysis. The 42 antimicrobials were effectively separated under the optimized experimental conditions; the target compounds exhibited linear working curves in the 0.25-5.0 mg/kg concentration range, with correlation coefficients (*r*) greater than 0.99. Limits of detection (LODs) for the 42 antimicrobials were determined from the signal-to-noise ratios (*S/N*) of their chromatographic peaks. LODs of 0.03-0.10 mg/kg were determined for the three matrices using 0.2-g samples and 10-mL test solution. Recoveries of 80.3-109.8%, with relative standard deviations (RSDs) of less than 9.8%, were obtained by determining three levels of each target analyte added to the three blank matrices; this process was repeated for six parallel samples. The developed method was used to analyze antimicrobials in commercially available disinfection products, with two sample batches testing positive. The established method is simple, accurate, precise, and suitable for the rapid screening and quantification of antimicrobials in disinfection products. This study provides powerful technical support for regulating the illegal addition of related antimicrobials to disinfection products.

抗微生物药物可以抑制致病微生物的生长和繁殖,缓解和治疗由致病微生物引起的皮肤或其他健康问题。抗微生物药物分为抗细菌药物、抗真菌药物、抗病毒药物等不同类别,包括唑类、磺胺类、四环素类、喹诺酮类等多种人工合成或天然化合物^[1,2]^。抗微生物药物需在医生的指导下使用,不当或过量使用抗微生物药物可能引起不良的皮肤反应,甚至损伤器官,例如,喹诺酮类药物可导致光敏反应、血糖紊乱和跟腱炎等不良反应,而对婴幼儿使用磺胺类药物时可能引发溶血性贫血、血红蛋白尿和高胆红素血症等问题^[3]^。长期使用抗微生物药物还会增加微生物对药物的耐药性,例如非白色念珠菌属对氟康唑产生耐药性^[4,5]^。此外,抗微生物药物进入环境后,可能抑制环境中微生物的活性,刺激病原菌产生抗药性,破坏原有的陆生或水生生态平衡。因此,消毒产品、化妆品等日用消费品的相关法规和标准明确禁止使用抗微生物药物^[6]^。不过,由于抗微生物药物可以增强产品消毒或抗抑菌作用,短期效果显著,因而在人体用消毒剂等消毒产品中非法添加抗微生物药物的情况时有发生^[7]^。因此,亟需建立检测方法,为监管提供技术支撑。

高效液相色谱-串联质谱(HPLC-MS/MS)具有高灵敏度、高选择性和强抗干扰能力等优点,是抗微生物药物检测方法建立和日常监督检验的常用方法。对抗微生物药物检测的研究主要集中在食品^[8⇓-10]^、化妆品^[11⇓-13]^、环境^[14⇓-16]^等基质中,目前对消毒产品中抗微生物药物的检测报道相对较少。朱峰等^[17]^建立了超高效液相色谱-串联质谱测定消毒产品中8类13种抗生素的分析方法,方法简单、可靠,重现性好。但其方法使用甲醇或乙腈直接提取膏霜样品,容易出现试样团聚不溶而难以分散的问题,缺乏净化手段导致仪器污染风险加大;方法采用内标法定量,在拓展检测药物种类时需要增加内标物种类,不易推广。王军淋等^[18]^采用甲酸水-乙腈提取和混合型阳离子固相萃取柱(MCX)净化实现了消毒产品中5种硝基咪唑类抗生素和17种磺胺类抗生素的测定,内标法定量,回收率为84.3%~121.2%,方法灵敏度高,选择性好,操作简单,适用于膏霜、水剂型消毒产品中22种违禁抗生素的检测。文献和现有消毒产品标准规范^[19⇓⇓-22]^中的方法检测的物质数量较少,不同种类的抗微生物药物难以在一个方法中同步测定。随着消毒产品类型逐渐丰富,尤其是凝胶、膏霜等剂型的广泛使用,已有方法提取效率有限或缺乏净化手段,影响目标物的定性和定量^[23,24]^。

本研究利用溶剂萃取和分散固相萃取的前处理方法,结合高效液相色谱-串联质谱,建立了膏霜、凝胶和水剂3类常见的消毒产品中42种禁用抗微生物药物的分析方法。42种抗微生物药物包括抗细菌药物、抗真菌药物和抗病毒药物,分别为7种磺胺类、10种喹诺酮类、3种林可酰胺类、5种四环素类、3种大环内酯类、8种唑类、3种嘌呤核苷类、1种呋喃类、1种非多烯类及1种类固醇类。本方法可为消毒产品中禁用抗微生物药物的快速风险筛查和日常监管提供技术支持。

## 1 实验部分

### 1.1 仪器、试剂与材料

QTRAP 4500高效液相色谱-串联质谱仪(美国SCIEX公司); Multi Reax涡旋混合器(德国Heidolph公司); 3-18K离心机(德国Sigma公司)。

实验用水为超纯水(英国ELGA公司);乙腈(色谱纯,安徽天地高纯溶剂有限公司),甲酸(分析纯,上海阿拉丁生化科技股份有限公司),无水硫酸钠(分析纯,国药集团化学试剂有限公司); QuEChERS dSPE EMR-Lipid分散固相萃取管(货号:5982-1010,安捷伦科技有限公司); 0.45 μm聚四氟乙烯滤膜(上海安谱实验科技股份有限公司)。

抗感染药物混合标准溶液(含甲硝唑等35种抗微生物药物,100 mg/L)、硝酸益康唑(纯度99.9%)、硝酸咪康唑(纯度99.9%)、磺胺嘧啶(纯度99.1%)、更昔洛韦(纯度98.0%)、阿昔洛韦(纯度92.5%±2.0%,含水5.7%,经仪器方法分析未检出其他抗微生物药物)、喷昔洛韦(纯度99.3%)、利巴韦林(纯度99.6%)购自上海安谱实验科技股份有限公司。

购买的抗感染药物混合标准溶液直接使用。更昔洛韦、阿昔洛韦、喷昔洛韦、利巴韦林标准品用水配制成1000 mg/L的标准储备液。硝酸益康唑、硝酸咪康唑、磺胺嘧啶用乙腈配制成1000 mg/L的标准储备液。使用时用乙腈稀释配制成标准中间液。系列标准工作溶液则用0.5%甲酸乙腈溶液配制。

### 1.2 样品前处理

称取0.2 g(精确到0.1 mg)均匀试样于15 mL塑料离心管中,加入2 mL水,涡旋振荡5 min。加入10 mL 0.5%甲酸乙腈溶液,涡旋振荡5 min。加入3 g无水硫酸钠,振荡,以8000 r/min转速离心5 min。吸取5 mL上清液,转移至预先加入1 mL水并振荡的QuEChERS dSPE EMR-Lipid净化管中,涡旋振荡5 min,以8000 r/min转速离心5 min。取上清液,加入2 g无水硫酸钠,振荡,静置。取清液过微孔滤膜后,供高效液相色谱-串联质谱分析。

### 1.3 高效液相色谱-串联质谱条件

色谱柱:Poroshell 120 EC-C18色谱柱(150 mm×3.0 mm, 2.7 μm);流动相A: 0.1%甲酸乙腈溶液,流动相B: 0.1%甲酸水溶液;流速:0.3 mL/min;进样体积:2 μL;柱温:35 ℃;梯度洗脱程序:0~3 min, 15%A; 3~9 min, 15%A~40%A; 9~12 min, 40%A~60%A; 12~14 min, 60%A~95%A; 14~20 min, 95%A; 20~21 min, 95%A~15%A; 21~23 min, 15%A。

离子源:电喷雾离子源(ESI);扫描方式:正离子扫描;检测模式:多反应监测(MRM)模式;气帘气压力:206.843 kPa(30.0 psi);喷雾电压:5500 V;雾化温度:500 ℃;喷雾气压力:344.750 kPa(50 psi);辅助气压力:344.750 kPa(50 psi);碰撞气压力:Medium。其他质谱参数见表1。

**表1:** 42种抗微生物药物的保留时间和质谱参数

No.	Analyte	*t*_R_/min	Precursor ion (*m/z*)	Declustering potential/V	Product ions (*m/z*)	Collision energies/eV
1	ganciclovir (更昔洛韦)	2.26	256.2	50	152.1^*^, 135.1	18, 40
2	ribavirin (利巴韦林)	2.29	245.0	50	112.9^*^, 95.8	15, 40
3	acyclovir (阿昔洛韦)	2.33	226.1	50	152.1^*^, 135.1	18, 40
4	penciclovir (喷昔洛韦)	2.45	254.1	50	152.1^*^, 135.1	25, 15
5	lincomycin (林可霉素)	3.84	407.2	90	359.0^*^, 126.0	25, 31
6	metronidazole (甲硝唑)	3.86	172.0	50	82.0^*^, 128.0	33, 19
7	furaltadone (呋喃它酮)	4.35	325.0	70	99.8^*^, 281.0	30, 15
8	sulfadiazine (磺胺嘧啶)	4.92	251.1	40	156.0^*^, 92.0	22, 38
9	enoxacin (依诺沙星)	5.13	321.0	80	231.8^*^, 302.8	47, 30
10	sulfapyridine (磺胺吡啶)	5.64	250.0	60	156.0^*^, 92.0	22, 35
11	norfloxacin (诺氟沙星)	5.71	320.0	80	232.8^*^, 275.8	32, 23
12	ofloxacin (氧氟沙星)	5.80	362.0	80	260.8^*^, 318.0	36, 26
13	fleroxacin (氟罗沙星)	5.96	370.0	80	268.8^*^, 325.8	35, 27
14	pefloxacin (培氟沙星)	6.07	334.0	80	232.8^*^, 289.8	34, 24
15	oxytetracycline (土霉素)	6.20	461.2	90	426.0^*^, 443.0	27, 18
16	ciprofloxacin (环丙沙星)	6.32	332.2	90	231.0^*^, 288.1	48, 24
17	sulfamerazine (磺胺甲嘧啶)	6.38	265.0	60	156.0^*^, 92.0	22, 38
18	tetracycline (四环素)	7.66	445.1	90	410.0^*^, 428.0	27, 18
19	enrofloxacin (恩诺沙星)	7.85	360.2	80	245.1^*^, 342.2	34, 29
20	sulfamethizole (磺胺甲二唑)	8.03	271.1	30	156.0^*^, 92.0	20, 36
21	sulfamethoxypyridazine (磺胺甲氧哒嗪)	8.21	281.1	60	156.0^*^, 92.0	22, 38
22	fluconazole (氟康唑)	8.79	307.1	80	238.1^*^, 220.0	21, 23
23	sarafloxacin (沙拉沙星)	8.92	386.0	80	299.1^*^, 342.1	37, 26
24	difloxacin (双氟沙星)	9.06	400.2	80	299.1^*^, 356.0	38, 26
25	moxifloxacin (莫西沙星)	9.48	402.2	90	261.1^*^, 364.5	32, 38
26	minocycline (米诺环素)	9.56	458.2	90	441.3^*^, 352.0	25, 41
27	clindamycin phosphate (克林霉素磷酸酯)	9.75	505.2	110	126.1^*^, 457.2	33, 27
28	sulfachloropyridazine (磺胺氯哒嗪)	9.84	285.0	60	155.9^*^, 92.0	19, 38
29	aureomycine (金霉素)	9.94	479.1	90	444.0^*^, 462.0	28, 24
30	azithromycin (阿奇霉素)	9.97	749.5	110	591.5^*^, 116.1	27, 70
31	clindamycin (克林霉素)	10.04	425.2	90	377.2^*^, 126.2	25, 31
32	doxycycline (多西霉素)	10.36	445.2	80	428.0^*^, 154.0	23, 35
33	sulfamethoxazole (磺胺甲噁唑)	10.52	254.1	60	156.0^*^, 107.9	21, 30
34	ketoconazole (酮康唑)	12.63	531.2	120	244.0^*^, 489.3	44, 42
35	clarithromycin (克拉霉素)	12.99	748.5	110	158.1^*^, 590.6	32, 27
36	roxithromycin (罗红霉素)	13.06	837.4	120	158.1^*^, 679.6	36, 30
37	clotrimazole (克霉唑)	13.50	277.1	100	165.0^*^, 215.0	24, 24
38	bifonazole (联苯苄唑)	13.64	311.2	40	243.1^*^, 165.0	15, 49
39	griseofulvin (灰黄霉素)	14.33	353.2	80	165.0^*^, 215.0	24, 24
40	econazole (益康唑)	14.60	381.0	30	125.0^*^, 193.0	31, 18
41	miconazole (咪康唑)	14.89	417.0	50	159.0^*^, 161.0	35, 32
42	spironolactone (螺内酯)	15.45	341.1	120	107.0^*^, 165.0	33, 85

* Quantitative ion.

## 2 结果与讨论

### 2.1 仪器条件优化

#### 2.1.1 质谱条件选择

采用电喷雾电离,将42种抗微生物药物的单一标准溶液或混合标准溶液(20 mg/L)注入离子源。在正离子扫描模式下对目标化合物进行一级质谱全扫描。目标化合物在正离子模式下均产生[M+H]^+^准分子离子峰。优化各个准分子离子峰的去簇电压,再进行二级质谱扫描。选取丰度较高且干扰较小的两个子离子分别作为定性和定量离子,其中响应较高的离子作为定量离子。分别优化两个子离子的碰撞能量。优化得到的质谱参数见表1。

#### 2.1.2 液相色谱条件选择

首先比较了有机相乙腈和甲醇对目标物洗脱及色谱峰的影响,乙腈对目标化合物的洗脱能力相对较强且色谱峰更为尖锐、良好,因而有机相优先选择乙腈。考虑到多种药物结构中含有磺胺、酰胺、羧酸等基团,预期流动相酸碱性可能会对待测组分的峰形、在质谱中的离子化强度等产生影响,因此比较目标物在酸性和中性流动相体系中的分离效果。考察了0.1%甲酸乙腈溶液-0.1%甲酸水溶液和0.1%乙酸乙腈溶液-0.1%乙酸水溶液作为流动相体系的分离效果。结果显示,流动相中含甲酸时色谱峰面积整体稍大于流动相中含乙酸时;采用乙腈-水、乙腈-乙酸铵水溶液、乙腈-氟化铵水溶液等中性流动相体系时,未测得10种喹诺酮类药物的色谱信号,测得的5种四环素类药物和3种大环内酯类药物的色谱峰面积比采用酸性流动相时减少30%~60%。因此最终确定以0.1%甲酸乙腈溶液-0.1%甲酸水溶液作为流动相,以提高部分抗微生物药物的检测灵敏度。

考虑待测药物成分多,极性差异较大,选择通用型反相色谱柱分离。对比Shim-pack XR-ODS Ⅲ(75 mm×2.0 mm, 1.6 μm)、Inertsil ODS-3(100 mm×2.1 mm, 3 μm)和Poroshell 120 EC-C18(150 mm×3.0 mm, 2.7 μm)3款色谱柱,结果表明,目标分析物响应基本一致,且能有效分离。考虑到利巴韦林、更昔洛韦等药物极性较大,过短的色谱柱对其保留和分离能力不足,最终选择柱长150 mm的Poroshell 120 EC-C18色谱柱。

42种抗微生物药物的总离子流色谱图见图1。

**图1:**
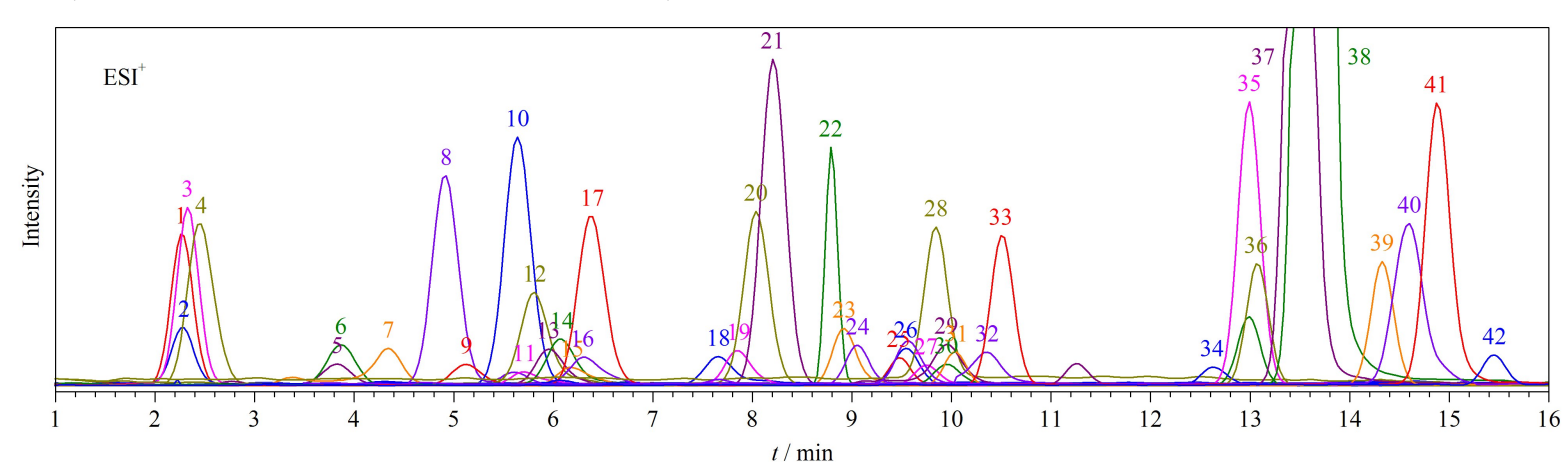
42种抗微生物药物(20 μg/L)的总离子流色谱图

### 2.2 前处理条件优化

#### 2.2.1 提取方式选择

本研究所涉及的抗微生物药物,大部分在乙腈中有较好的溶解性。参考文献[11,12,17],首先选择直接用乙腈提取的处理方式。由于膏霜基质常使用脂质、蜡质、表面活性剂和保湿润滑剂等成分,而凝胶基质常使用水性聚合物、表面活性剂等成分,因而在用10 mL乙腈提取一部分膏霜和凝胶类样品时,出现样品团聚而不易分散的情况。

为了充分分散样品,实验考察了采用水-有机溶剂进行液液萃取的可行性^[13]^。使用3 mL饱和氯化钠溶液和5 mL乙腈来处理0.2 g加标膏霜和凝胶试样。结果显示,更昔洛韦、利巴韦林、克林霉素磷酸酯等在水中具有一定的溶解性,与其他抗微生物药物不同,在乙腈层中分配量较少,因而使用饱和氯化钠溶液-乙腈萃取体系难以同步提取42种抗微生物药物。

因此后续采取水辅助分散的处理方式,对于团聚不溶的样品,预先加入2 mL水,再用10 mL乙腈提取。结果显示,水可以有效分散样品,有助于乙腈进一步提取样品中的抗微生物药物。比较用乙腈提取和先用水分散再加乙腈提取两种方式的提取效果(图2),用水辅助分散的方式可以提高乙腈对各抗微生物药物的提取效率。

**图2:**
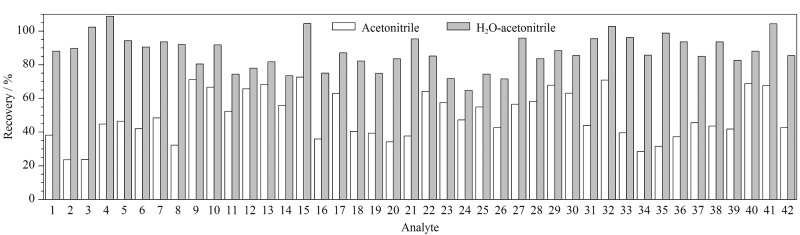
膏霜基质中42种抗微生物药物在不同提取溶剂下的加标回收率

#### 2.2.2 提取溶剂选择

由于多种目标药物的结构中含有可电离基团,且基质中添加表面活性剂等水溶性成分也往往含有可电离基团,因而考察了提取溶剂中添加有机酸对提取效率的影响。

分别用乙腈和0.5%甲酸乙腈提取水预分散的加标膏霜试样,测定并计算各抗微生物药物含量。结果显示,添加甲酸可以有效提高氧氟沙星等10种喹诺酮类药物的提取效率,用乙腈提取喹诺酮类药物的加标回收率仅为用0.5%甲酸乙腈提取时的57.4%~87.1%。另外,采用甲酸乙腈溶液提取时提取液相对澄清,可能是有机酸有利于基质中其他物质的析出和沉淀。因此,选择0.5%甲酸乙腈溶液作为提取溶剂。

#### 2.2.3 净化方式选择

对于溶解在乙腈中的基质成分,选择通过固相萃取方式净化。由于各类抗微生物药物的基团、极性等分子结构特征存在较大差异,常规的Silica填料、C18填料、NH_2_填料(氨丙基)、PSA填料等仅能对其中的几种或几类物质有保留,容易在上样和淋洗步骤中造成目标物损失或丢失。因此选择用吸附剂直接去除杂质的方法。同时选择操作更为简单便捷的分散固相萃取方式代替固相萃取小柱净化方式。考虑到基质成分具有长烷烃链的特点,选用增强型脂质去除EMR-Lipid净化剂。该净化剂通过空间位阻和亲疏水性协同作用,对于C5及以上碳链化合物具有极强的选择性吸附,可以除去干扰脂质等基质杂质而不吸附目标物^[25]^;净化剂已应用于食用油、肉类、乳制品等富含脂肪、磷脂基质中农药、兽药残留和污染物的测定,也应用于洗衣液、洗衣皂等含大量表面活性剂基质中小分子化合物的测定^[26]^。在净化过程中,为减少活化净化剂的水对乙腈溶液中各化合物定量的影响,净化剂处理后用无水硫酸钠除去活化时加入的水。

考察EMR-Lipid净化剂对提取液的净化效果和对目标物回收率的影响。将净化剂处理前后的空白样品提取液用氮气吹干,观察残留杂质的情况。结果显示,净化剂可以有效降低提取液中杂质的量(图3a)。分析净化剂处理前后空白样品加标提取液中目标物的回收率。结果显示,净化有效提高了嘌呤核苷类、喹诺酮类、大环内酯类等多种目标物的回收率(图3b),推测是净化剂降低了基质对目标物信号的抑制作用。

**图3:**
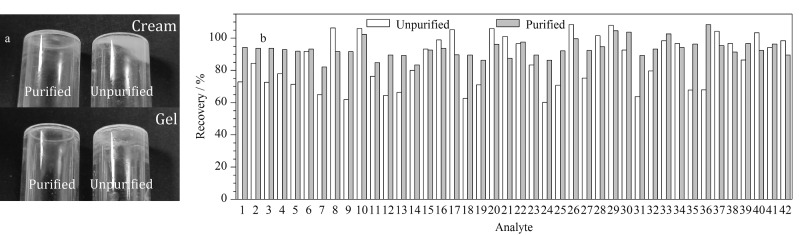
净化剂对(a)净化效果和(b)抗微生物药物加标回收率的影响

### 2.3 基质效应

膏霜和凝胶等复杂基质中同时被提取出的杂质可能会影响目标物在仪器中的信号强度,产生基质效应(ME)^[11]^,基质效应会影响分析结果的准确性。用基质匹配标准曲线斜率*k*_1_和溶剂标准曲线斜率*k*_2_的比值评价基质效应。当ME为100%~120%时,显示较弱的基质增强效应;当ME>120%时,显示较强的基质增强效应;当ME为80%~100%时,显示较弱的基质抑制效应;当ME<80%时,显示较强的基质抑制效应。从表2结果可知,膏霜基质对各种目标化合物的基质效应为82.6%~104.7%,基质效应对各抗微生物药物的定量影响较小。

**表2:** 42种抗微生物药物的线性范围、相关系数、检出限、定量限和基质效应(膏霜)

No.	Analyte	Linear range/(mg/kg)	*r*	LOD/(mg/kg)	LOQ/(mg/kg)	ME/%
1	ganciclovir	0.25-5.0	0.99759	0.10	0.25	97.6
2	ribavirin	0.25-5.0	0.99841	0.03	0.10	98.6
3	acyclovir	0.25-5.0	0.99804	0.10	0.25	96.9
4	penciclovir	0.25-5.0	0.99763	0.10	0.25	93.7
5	lincomycin	0.25-5.0	0.99813	0.10	0.25	93.6
6	metronidazole	0.25-5.0	0.99751	0.10	0.25	95.4
7	furaltadone	0.25-5.0	0.99865	0.10	0.25	87.2
8	sulfadiazine	0.25-5.0	0.99986	0.10	0.25	93.6
9	enoxacin	0.25-5.0	0.99807	0.10	0.25	91.5
10	sulfapyridine	0.25-5.0	0.99750	0.10	0.25	85.9
11	norfloxacin	0.25-5.0	0.99850	0.10	0.25	82.6
12	ofloxacin	0.25-5.0	0.99709	0.10	0.25	86.7
13	fleroxacin	0.25-5.0	0.99868	0.10	0.25	87.3
14	pefloxacin	0.25-5.0	0.99972	0.10	0.25	82.9
15	oxytetracycline	0.25-5.0	0.99869	0.10	0.25	103.1
16	ciprofloxacin	0.25-5.0	0.99734	0.10	0.25	91.3
17	sulfamerazine	0.25-5.0	0.99973	0.10	0.25	83.8
18	tetracycline	0.25-5.0	0.99789	0.10	0.25	103.6
19	enrofloxacin	0.25-5.0	0.99791	0.10	0.25	84.9
20	sulfamethizole	0.25-5.0	0.99841	0.10	0.25	93.1
21	sulfamethoxypyridazine	0.25-5.0	0.99679	0.10	0.25	86.7
22	fluconazole	0.25-5.0	0.99567	0.10	0.25	96.8
23	sarafloxacin	0.25-5.0	0.99993	0.10	0.25	84.7
24	difloxacin	0.25-5.0	0.99929	0.10	0.25	87.2
25	moxifloxacin	0.25-5.0	0.99808	0.10	0.25	94.3
26	minocycline	0.25-5.0	0.99926	0.10	0.25	98.7
27	clindamycin phosphate	0.25-5.0	0.99919	0.10	0.25	95.3
28	sulfachloropyridazine	0.25-5.0	0.99933	0.10	0.25	103.4
29	aureomycine	0.25-5.0	0.99717	0.10	0.25	104.2
30	azithromycin	0.25-5.0	0.99789	0.03	0.10	100.4
31	clindamycin	0.25-5.0	0.99875	0.10	0.25	91.3
32	doxycycline	0.25-5.0	0.99756	0.10	0.25	102.7
33	sulfamethoxazole	0.25-5.0	0.99703	0.10	0.25	96.5
34	ketoconazole	0.25-5.0	0.99794	0.10	0.25	97.4
35	clarithromycin	0.25-5.0	0.99666	0.03	0.10	104.7
36	roxithromycin	0.25-5.0	0.99856	0.03	0.10	98.7
37	clotrimazole	0.25-5.0	0.99976	0.10	0.25	95.6
38	bifonazole	0.25-5.0	0.99651	0.03	0.10	92.7
39	griseofulvin	0.25-5.0	0.99621	0.10	0.25	104.7
40	econazole	0.25-5.0	0.99653	0.10	0.25	97.3
41	miconazole	0.25-5.0	0.99861	0.10	0.25	96.9
42	spironolactone	0.25-5.0	0.99747	0.10	0.25	102.8

### 2.4 方法学验证

#### 2.4.1 线性范围、检出限与定量限

配制系列浓度的混合标准工作溶液,按照优化的条件进行测定,绘制标准工作曲线,计算相关系数(*r*)。当取样量0.2 g、定容体积10 mL时,42种抗微生物药物在0.25~5.0 mg/kg范围内线性良好(见表2)。

取基质效应较大的膏霜类空白样品,添加目标分析物,制备一系列低浓度加标试样,分析试样并计算各药物的色谱峰信噪比(*S/N*)。以*S/N*≥3的最低添加含量作为检出限(LOD),以*S/N*≥10的最低添加含量作为定量限(LOQ)。当取样量0.2 g、定容体积10 mL时,42种抗微生物药物检出限和定量限分别为0.03~0.10 mg/kg和0.10~0.25 mg/kg(见表2)。

#### 2.4.2 准确性和精密度

在膏霜、凝胶和水剂空白样品中添加标准溶液以制备0.25、1.0和5.0 mg/kg 3个水平的阳性样品,每个水平平行试验6次。按建立的方法测定样品,计算各水平下平行样品的平均回收率和相对标准偏差。膏霜基质中抗微生物药物的平均回收率为80.3%~99.8%,相对标准偏差≤9.8%;凝胶基质中抗微生物药物的平均回收率为82.8%~109.8%,相对标准偏差≤8.3%;水剂基质中抗微生物药物的平均回收率为86.4%~105.7%,相对标准偏差≤6.7%。3种基质中,方法精密度较高,回收率良好。膏霜基质中复杂物质可能是导致整体回收率和相对标准偏差较凝胶和水剂欠佳的原因。

### 2.5 实际样品检测

将建立的方法用于市售样品中抗微生物药物的测试,膏霜类消毒产品40批次、凝胶类消毒产品20批次、水剂类消毒产品20批次。在1批次(20210118)膏霜类消毒产品中检出酮康唑,含量为0.82 mg/kg;在1批次(230501)膏霜类消毒产品中检出咪康唑,含量为0.34 mg/kg;两种被检出抗微生物药物的MRM色谱图参见图4。方法可以有效用于消毒产品中相关药物的测定。

**图4:**
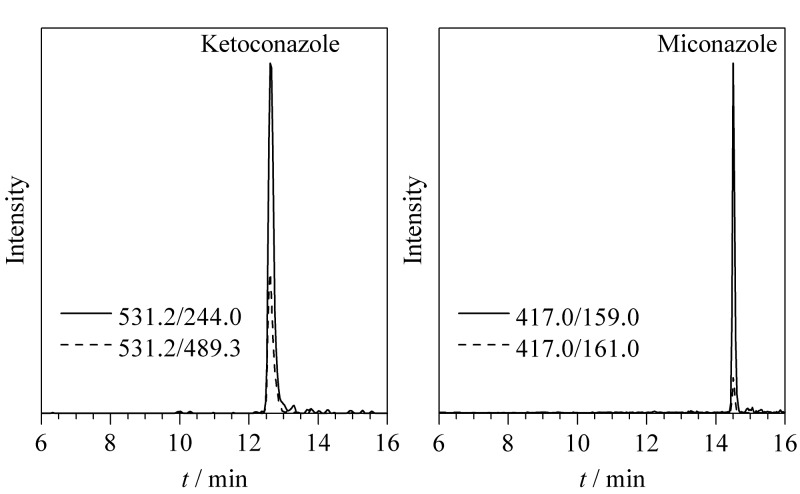
膏霜样品中检出药物的MRM色谱图

### 2.6 与文献方法比较

与文献中报道方法相比(见表3),本方法可同时测定42种抗微生物药物,拓展了检测药物的种类,提高了日常检测效率。采用分散固相萃取净化操作,步骤简单,减少基质效应影响,有效提高回收率。本方法采用外标法,相较于内标法,可以降低实验成本,同时可以减少内标配制等实验步骤。

**表3:** 本方法与文献方法的比较

Number of antimicrobials	Extraction solvents	Purification material	LODs/(mg/kg)					Quantitative method	Recovery/ %	Ref.
			Metroni- dazole	Aureomy- cine	Sulfapyri- dine	Sulfamet- hazine	Norfloxa- cin			
13	acetonitrile or methanol	-	0.015	0.025	0.010	-	0.015	internal standard method	71.2- 130.4	[17]
22	2% formic acid aqueous solution-acetonitrile (3∶2, v/v)	MCX	0.060	-	-	0.060	-	internal standard method	84.3- 121.2	[18]
42	water, and 0.5% formic acid acetonitrile	EMR-Lipid	0.10	0.10	0.10	0.10	0.10	external standard method	80.3- 109.8	this work

-: not mentioned.

## 3 结论

本研究建立了分散固相萃取-高效液相色谱-串联质谱同时测定消毒产品中42种抗微生物药物的方法。采用水辅助分散再用乙腈提取的方式,有效分散膏霜、凝胶基质。采用操作简单的分散固相萃取,净化剂能有效除去样品基质中的主要干扰物质。为了应对不断更新的基质配方,可以进一步尝试组合不同的净化剂。该方法简单快捷,准确性高,重复性好,适合消毒产品中抗微生物药物的快速筛查和定量,为监管消毒产品中相关药物的非法添加提供了有力的技术支持。
